# Advancements and Challenges in Handwritten Text Recognition: A Comprehensive Survey

**DOI:** 10.3390/jimaging10010018

**Published:** 2024-01-08

**Authors:** Wissam AlKendi, Franck Gechter, Laurent Heyberger, Christophe Guyeux

**Affiliations:** 1 CIAD, UMR 7533, UTBM, F-90010 Belfort, France; franck.gechter@utbm.fr; 2 FEMTO-ST Institute/RECITS, UMR 6174 CNRS, UTBM, F-90010 Belfort, France; laurent.heyberger@utbm.fr; 3 FEMTO-ST Institute/DISC, UMR 6174 CNRS, Université de Franche-Comté, F-90016 Belfort, France; christophe.guyeux@univ-fcomte.fr

**Keywords:** handwritten text recognition (HTR), HTR datasets, machine learning, Belfort civil registers of births, ICFHR, ICDAR

## Abstract

Handwritten Text Recognition (HTR) is essential for digitizing historical documents in different kinds of archives. In this study, we introduce a hybrid form archive written in French: the Belfort civil registers of births. The digitization of these historical documents is challenging due to their unique characteristics such as writing style variations, overlapped characters and words, and marginal annotations. The objective of this survey paper is to summarize research on handwritten text documents and provide research directions toward effectively transcribing this French dataset. To achieve this goal, we presented a brief survey of several modern and historical HTR offline systems of different international languages, and the top state-of-the-art contributions reported of the French language specifically. The survey classifies the HTR systems based on techniques employed, datasets used, publication years, and the level of recognition. Furthermore, an analysis of the systems’ accuracies is presented, highlighting the best-performing approach. We have also showcased the performance of some HTR commercial systems. In addition, this paper presents a summarization of the HTR datasets that publicly available, especially those identified as benchmark datasets in the International Conference on Document Analysis and Recognition (ICDAR) and the International Conference on Frontiers in Handwriting Recognition (ICFHR) competitions. This paper, therefore, presents updated state-of-the-art research in HTR and highlights new directions in the research field.

## 1. Introduction

**Figure 1:**
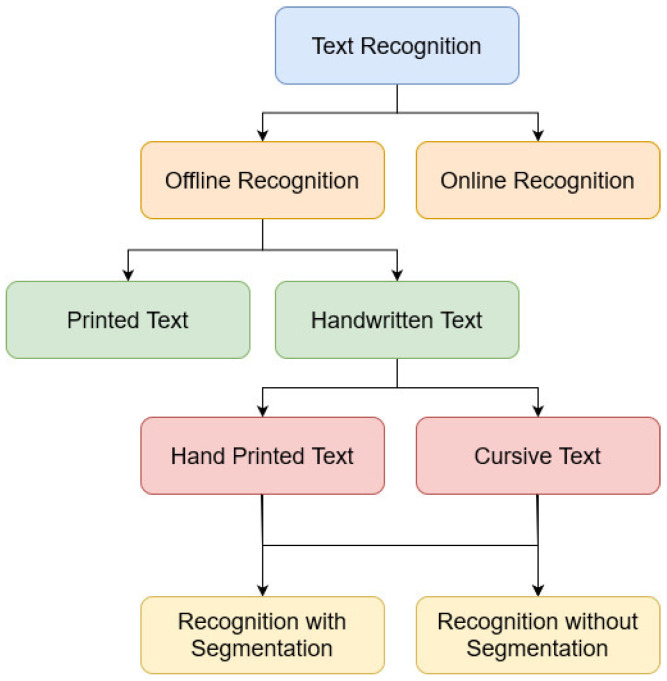
Classification of handwritten text recognition systems.

In recent years, handwritten text recognition has become one of the most critical research fields of pattern recognition. Many researchers proposed techniques to facilitate the possibility of transcribing historical archives [1], medical prescriptions [2], general forms, and any modern documents, through spatial (offline) or temporal (online) process [3]. Figure 1 shows the classification of the text recognition systems. Transcription involves automatically transforming a source’s handwritten text within a digital image into its machine text representation. Optical Character Recognition (OCR) [4] represents the cornerstone technique of this field. It consists of two main phases: firstly, detecting the text by segmenting it into small patches. Secondly, recognizing the contents of the patches in order to be transcribed into machine-coded text, the first and simplest OCR developed in [5] for the recognition of Latin numerals.

Fortunately, HTR systems have incredibly improved since utilizing the Hidden Markov Model (HMM) for text recognition and handcrafted features [6,7,8]. However, the recognition results of HMMs are still poor due to some drawbacks in the model, such as memorylessness [9] and the manual feature selection process. Researchers overcome these problems by proposing hybrid systems that combine additional architecture with HMM, for instance, HMM with Gaussian mixture emission distributions (HMM-GMM) [10], HMM with Convolutional Neural Network (CNN) [11], or HMM with Recurrent Neural Network (RNN) [12] which have significantly improved the outcomes.

Nowadays, systems can analyze document layouts and recognize letters, text lines, paragraphs, and whole documents. Arguably, these modern systems can recognize different handwritten styles in French, Arabic, Urdu, Chinese, and other languages. It includes the utilization of machine learning techniques such as Convolutional Neural Networks (CNN) [13], Recurrent Neural Networks (RNN) [14], Convolutional Recurrent Neural Networks (CRNN) [15], Gated-CNN [16], Multi-Dimensional Long Short-Term Memory Recurrent Neural Networks (MDLSTM-RNNs) [17]. Despite the many significant advancements in the past few years, there are still many challenges that need to be addressed.

Civil registers of births, or vital records, were created during the French Revolution. Since 1792, for all French men and women, regardless of their religion, all births that occur in each commune have been recorded, whether or not the parents reside in the commune concerned.

The study of the records of the commune of Belfort is very important for historical reasons: Belfort is a hapax in the French demographic landscape of the 19th century because, following the defeat of France against Prussia in 1871, this city experienced a mushrooming growth due to the influx of a large number of Alsatians, especially Mulhousians, who either decided to remain French after the annexation of Alsace–Lorraine by the newly created Germany or migrated to Belfort to continue working for the textile and mechanical industries that massively settled in the city after 1871. This city thus constitutes a privileged observatory of three major changes in characteristics of the European 19th century, in terms of urban growth, migration, and the sexualization of social relations. The historical issues that can be grasped from birth records alone are therefore numerous and important (study of midwives, concubinage, age at first child, inter-birth period and contraception, births outside of marriage, recognition of children, choice of witnesses for the declaration, choice of the child’s first name and home or hospital births). To effectively analyze and address these historical questions, it is essential to establish a comprehensive knowledge database that will facilitate their exploration and resolution. This process involves transcribing the archive contents through two approaches. The first is manual transcription by humans. However, this approach is expensive and time-consuming. The second is automatic transcription utilizing computer science methods.

### 1.1. Purpose and Contributions

Our intention is to provide an overview of existing handwritten text recognition models and highlight the commercially usable systems to acquire insights into the achievements in both modern and historical document recognition on a global scale, with a specific focus on the French language. This background will allow researchers to determine which novel techniques or combinations of techniques should be explored in order to transcribe the hybrid (printed and handwritten) text, as exemplified in the Belfort civil registers of births.

This paper also provides researchers with an up-to-date survey of state-of-the-art models. The survey encompasses research papers at four HTR levels: word, text line, paragraph, and page. Furthermore, a list of 14 publicly available datasets has been summarized for French and other languages, including the datasets used in the International Conference on Frontiers of Handwriting Recognition (ICFHR) and the International Conference on Document Analysis and Recognition (ICDAR) competitions.

### 1.2. Selection Methodology

We followed specific criteria in selecting the publications, including those that introduce novel techniques, are accessible, and widely cited. Our focus was primarily on recent studies published in reputable scientific journals and conference proceedings to ensure the reliability and credibility of the information. Additionally, we also took into consideration the publication year for both international and French-language HTR systems over a time span ranging from 2012 to 2023.

Furthermore, we have selected datasets presented at the International Conference on Document Analysis and Recognition (ICDAR) and the International Conference on Frontiers of Handwriting Recognition (ICFHR). Additionally, our aim was to present the most well-known datasets for various languages, focusing on historical document image datasets and those specific to the French language. This selection spans from 1999 to 2021. Also, we selected and presented some HTR commercial systems based on their offering of free trials for text recognition.

These approaches allowed for providing valuable insights into the advancements and challenges within this domain. The publications and datasets were selected using Google Scholar and Web of Science.

The rest of this paper is organized as follows: Section 2 provides an overview of the literature on HTR models and recent achievements in recognizing handwritten text, specifically focusing on the French language. In Section 3, a summary of the publicly available datasets is presented. Section 4 is devoted to providing a summary of the state-of-the-art experimental results. Then in Section 5, a discussion on the current work related to the French language is proposed. In Section 6, we present suggestions for future research directions. Finally, the conclusion is drawn in Section 7.

## 2. Literature Review

### 2.1. State-of-the-Art Recent Surveys

Several surveys have been published to advance the field and address its challenges. Authors of [18,19,20] presented a systematic literature review that summarized and analyzed the research articles conducted on character recognition of handwritten text documents across six languages in order to highlight research directions. Others conducted a survey on automated approaches in character recognition of historical documents [21,22]. The studies covered historical manuscripts in various languages, including Indian, Kurdish-Latin, Arabic, ancient Chinese, and others. They also summarized the techniques used for data pre-processing and the types of datasets utilized. Additionally, in [23], authors conducted a survey on state-of-the-art applications, techniques, and challenges in Arabic language character recognition. In [24], authors surveyed the challenges of the recognition and classification of named entities in various historical resources and languages, including the French language. Additionally, they discussed the employed approaches in the named entity recognition (NER) field and highlighted directions for future developments.

In [25], authors introduced an open database of historical handwritten documents that are fully annotated in the Norwegian language. To assess the performance of state-of-the-art HTR models on their dataset, they conducted a systematic survey of open-source HTR models, including twelve models with different characteristics, Their study highlighted the best performing technique and suggested a combination of different models to further improve performance. In [26], authors present a systematic literature review of image datasets for historical document image analysis at two scales: document classification and layout structure or content analysis. The research aims to assist researchers in identifying the most suitable datasets for their techniques and to advance the field of historical document image analysis. Similarly, others focused on the databases and benchmarks of this field [27].

On the other hand, authors of [28] surveyed the major phases of historical document digitization process, focusing on the standard algorithms, tools, and datasets within the field. Their research highlighted the critical importance of transcription accuracy as a prerequisite for meaningful information retrieval in archival documents. In contrast, authors of [29] focused on the feature extraction phase in handwritten Arabic text recognition.

Additionally, in [30], authors presented a critical study of various document layout analysis (DLA) techniques aimed at detecting and annotating the physical structure of documents. The survey highlighted the different phases of DLA algorithms, including preprocessing, layout analysis strategies, post-processing, and performance evaluation. This research serves as a base step toward achieving a universal algorithm suitable for all types of document layouts.

In the study [31], authors discussed the importance of separating machine-printed texts and handwritten texts in hybrid-form documents to enhance the overall system accuracy. The discussion involved techniques employed for the separation process based on feature extraction methods for three categories: structural and statistical features, gradient features, and geometric features.

This research establishes an updated state-of-the-art survey of HTR systems, different languages datasets, HTR competitions, and HTR commercial systems of the French language and other international ones.

### 2.2. Handwritten Text Recognition Workflow

**Figure 2:**
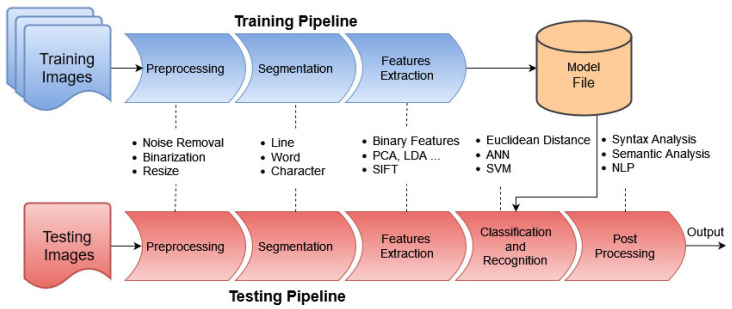
Handwritten text recognition general pipeline.

The workflow of handwritten text recognition includes classical image processing approaches and deep learning approaches. Figure 2 illustrates the general pipeline of the HTR workflow.

#### 2.2.1. Image Digitization

It is the process of transforming a handwritten text image into an electronic form using various devices such as scanners and digital cameras. This form of the image can be used as the input to the pre-processing stage.

#### 2.2.2. Pre-Processing

Pre-processing is the initial stage in enhancing digital images, it involves several key processes such as:

- Binarization: This process involves converting digital images into binary images consisting of dual collections of pixels in black and white (0 and 1). Binarization is valuable for segmenting the image into foreground text and background.
- Noise removal: This process involves eliminating unwanted pixels from the digitized image that can affect the original information. This noise may originate from the image sensor and electronic components of a scanner or digital camera. Various methods have been proposed for noise removal or reduction, such as Non-local means [32] and Anisotropic diffusion [33], as well as filters like Gaussian, Mean, and Median filters.
- Edges detection: This process involves identifying the edges of the text within the digitized image using various methods such as Sobel, Laplacian, Canny, and Prewitt edge detection.
- Skew detection and correction: Skew refers to the misalignment of text within a digital image. In other words, it indicates the amount of rotation needed to align the text horizontally or vertically. Various methods for skew detection and correction have been proposed to address this objective, such as Hough transforms and clustering.
- Normalization: This process involves reducing the shape and size variation of digital images. Additionally, it scales the input image features to a fixed range (e.g., between 0 and 1), while maintaining the relationship between these features. This process plays a valuable role in the training stage of deep learning models.

#### 2.2.3. Segmentation

This process involves dividing the handwritten text image into characters, words, lines, and paragraphs, often utilizing the pixel characteristics within the image. Several methods have been developed to conduct the segmentation process, including threshold methods, region-based methods, edge-based methods, watershed-based methods, and clustering-based methods. The segmentation stage is considered as one of the most crucial steps that can significantly improve the accuracy of HTR models [34].

#### 2.2.4. Feature Extraction

This process involves extracting specific information from the images that precisely represents the image, with the goal of reducing the size of high-dimensional data. The feature extraction process aims for higher discriminating power and control overfitting problems within HTR models. However, it may lead to a loss of data interpretability [35]. The most popular techniques for feature extraction include Principal Component Analysis (PCA), Linear Discriminant Analysis, and Independent Component Analysis (ICA). The accuracy of HTR systems is highly dependent on the choice of feature extraction techniques.

#### 2.2.5. Classification

This process involves deciding the class membership in the HTR system. The decision-making criteria depend on comparing the input feature with a predefined pattern to identify the most suitable matching class for the input. Two key methods can be employed in this stage. First, the template-based method [36] calculates the correlation between the input and the predefined pattern. Second, the feature-based method [37] utilizes the extracted features from the input for classification.

#### 2.2.6. Post-Processing

This stage aims to improve the results of the classification stage and enhance the overall accuracy of the HTR models. It involves output error correction using various techniques such as Dictionary lookup and statistical approaches. However, this stage is not compulsory in the HTR models development process.

### 2.3. Advancements in Handwritten Text Recognition: A State-of-the-Art Overview

Languages, by nature, vary in their letter shapes and word connections. The process of recognizing handwritten text is complicated due to many impediments such as the variety of writing style, documents poor quality, noise, spots on paper, and text alignment.

Researchers have proposed several models for recognizing language scripts using different architectures, including Convolutional Neural Network (CNN), Convolutional Recurrent Neural Network, sequence-to-sequence Transformer, Bidirectional Long Short-Term Memory (BLSTM), and others.

In a recent study, a Deep Learning (DL) system using two CNN architectures, named HMB1 and HMB2, was employed to recognize handwritten Arabic characters [38]. The models were trained using a complex Arabic handwritten characters dataset (HMBD), as well as CMATER [39,40] and AIA9k [41] datasets. The model demonstrated a significant accuracy rate when testing HMB1 on HMBD, which further improved when a data augmentation process was applied. Additionally, CMATER and AIA9k datasets were utilized to validate the generalization of the model. In the same context, two different architectures, namely the transformer transducer and sequence-to-sequence transformer, have been established in [42]. These architectures were evaluated using the KFUPM Handwritten Arabic TexT (KHATT) dataset [43,44,45]. Several pre-processing steps, such as text lines rotation and elimination of empty spaces, were performed. An attention mechanism was then applied to reduce the model complexity and achieve satisfactory performance in terms of accuracy and latency, surpassing the previous literature on the KHATT benchmark. Similarly, in [46], a light encoder–decoder transformer-based architecture was introduced for handwriting text recognition, while in [47], an end-to-end approach with pre-trained image transformer and text transformer models for text recognition at the word level was proposed. More survey articles and recent research works for recognizing handwritten text can be found in [17,48,49,50,51,52].

Other researchers introduced Attention-Based Fully Gated CNN supported by multiple bidirectional gated recurrent units (BGRU), with a Connectionist Temporal Classification (CTC) model to predict the sequence of characters [53]. They evaluated this method using five well-known datasets within the HTR community, which encompassed Institut für Informatik und Angewandte Mathematik (IAM) [54], Saint Gall [55], Bentham [56], and Washington [57] for English, as well as the Russian–Kazakh dataset (HKR) [58]. The method achieved a remarkably high recognition rate with minimal parameter usage when applied to the latter dataset.

Furthermore, a novel approach was introduced that combines depthwise convolution with a gated-convolutional neural network and bidirectional gated recurrent unit [59]. This technique effectively reduces the total number of parameters while simultaneously enhancing the overall performance of the model. Authors of [15,60] presented Convolutional Recurrent Neural Network (CRNN) architecture; the latter utilized CRNN for handwriting recognition as an encoder for the input text lines while utilizing a Bidirectional Long Short-Term Memory (BLSTM) network followed by a fully CNN as a decoder to predict the sequence of characters. IAM and Reconnaissance et Indexation de données Manuscrites et de fac similÉS (RIMES) [61] with the newly created dataset (EPARCHOS) [60] that includes historical Greek manuscripts have been used in the evaluation process of the proposed architecture. Experiments produced improved results compared with the other state-of-the-art methods, especially on the RIMES dataset. Table 1 summarizes the articles based on the architectures used at different HTR levels for different international languages.

Conversely, many researchers evaluated various models on the French handwritten text. In the competition organized at ICDAR2011 [63] using the RIMES dataset, authors of [64] presented a combination of three techniques of multi-word recognition. Firstly, Grapheme-based Multilayer perceptron (MLP)-HMM was used to decompose the words into letters. Secondly, sliding window Gaussian mixture HMM was used to model the letters with a HMM model using a Gaussian distribution mixture for the observation probabilities. Finally, training a MDLSTM-RNN model using raw values of pixels represents words as inputs. The system recorded an advanced recognition rate on the RIMES dataset for both word and multi-word recognition tasks. Similarly, an MDLSTM-RNN-CTC model using Graphics processing unit (GPU)-based implementation was proposed in [65] to decrease the model training time by processing the input text lines in a diagonal-wise mode, while in [66], authors applied the concept of curriculum learning to the MDLSTM-Convolution Layers-Fully Connected Network (FCN)-CTC model in order to enhance the learning process speed. Additionally, an attention-based RNN-LSTM architecture was proposed in [67]. This architecture was evaluated using the RIMES 2011 dataset. Another research study [66] on datasets such as IAM, RIMES, and OpenHaRT demonstrated significant improvement rates as a result of applying the curriculum learning concept to their model. However, due to alignment issues commonly observed in attention models caused by the recurrence alignment operation, authors of [68] introduced a decoupled attention network (DAN). The DAN is an end-to-end text recognizer that comprises three components. Firstly, a feature encoder is utilized to extract visual features from the source image. Secondly, a convolutional alignment module is employed. Finally, a decoupled text decoder is used for the prediction stage. The model has undergone numerous experiments using IAM and RIMES datasets, achieving effectiveness and merit recognition rates.

Furthermore, in [69], authors presented a system based on recurrent neural networks with weighted finite state transducers and an automatic mechanism for preparing annotated text lines to facilitate the model training process. The model used to decode sequences of characters or words on Maurdor [70] dataset. In the same context, the work was extended to text line recognition; the approach depends on segmenting the text line into words classified based on the confidence score into an anchor and non-anchor words (AWs and NAWs), AWs were equated to the BLSTM outputs, while dynamic dictionaries were created for NAWs by exploiting web resources for their character sequence. Finally, text lines were decoded using dynamic dictionaries [71].

Additionally, authors of [72] introduced a combination of a deep convolutional network with a recurrent encoder–decoder network to predict the sequence of characters at the word level within IAM and RIMES datasets images. Furthermore, in [73], authors combined CTC approaches with Sequence-To-Sequence (S2S) model to improve the recognition rate on the text line level, they developed the model based on a CNN as a visual backbone, BLSTM as encoder, and a Transformer used for character-wise S2S decoding. The evaluation process using IAM, RIMES, and Staatsarchiv des Kantons Zürich (StAZH) datasets shows competitive recognition results with 10-20 times fewer parameters.

Authors of [74] claimed that Multidimensional Long Short-Term Memory networks might not be necessary to attain good accuracy for HTR due to expensive computational costs. Instead, they suggested an alternative model that relies only on convolutional and one-dimensional recurrent layers. Experiments were carried out using IAM and RIMES 2006 datasets and achieved faster performance with equivalent or better results than MDLSTM models. Similarly, a multilingual handwriting recognition model that leverages a convolutional encoder for input images and a bidirectional LSTM decoder was presented to predict character sequences [75].

Additionally, as neural networks require large data amount in the training stage in order to improve the accuracy of recognition, and because the process of creating transcription data is expensive and time-consuming, authors of [76] presented a model architecture that aims to automatically transcribe Latin and French medieval documentary manuscripts produced between the 12th and 15th centuries based on a CRNN network with a CTC loss; the model has been trained depending on The Alcar-HOME database, the e-NDP corpus, and the Himanis project [77].

Recently, some research studies proposed an end-to-end architecture to recognize handwriting text at the paragraph level [78,79,80], the latter study introduced an end-to-end transformer-based approach for text recognition and named entities from multi-line historical marriage records images in the competition ICDAR 2017 (Esposalles [81] and French Handwritten Marriage Records (FHMR) [82]), while in [78], an end-to-end recurrence-free fully convolutional network named Simple Predict & Align Network (SPAN) was presented, performing OCR on RIMES, IAM, and READ 2016 at the paragraph level. Additionally, in [79], Vertical Attention Network (VAN), a novel end-to-end encoder–decoder segmentation-free architecture using hybrid attention was introduced.

Furthermore, research in [62,83,84] introduced segmentation-free document level recognition. Authors of [62] proposed a simple neural network module (OrigamiNet) that can augment with any fully convolutional single line text recognizer to convert it into a multi-line/full page recognizer. They conducted model experiments on the ICDAR2017 [85] competition and IAM datasets to demonstrate the applicability and generality of the proposed module, while authors of [83,84] presented Document Attention Network (DAN), an end-to-end architecture for full document text and layout recognition. DAN trained using a pre-defined labeling module for transcribing pages by tags style equivalent to Extensible Markup Language (XML) style aims to process the physical and geometrical information with language supervision only and reduce the annotation costs. The model predicts the document’s text lines in parallel after determining the first character of each line. It has undergone numerous experiments using RIMES 2009 and READ 2016 and showed highly beneficial recognition rates on a text line, paragraph, and document level. Table 2 summarizes articles based on the architectures used at different HTR levels for the French language.

More methodologies on the recognition of French and other languages on different prediction levels can be found in: characters [86,87], words [88,89,90], lines [91,92], paragraphs [93,94], and pages [95,96].

**Table 2:** State-of-the-art architectures utilized for handwritten text recognition of French language datasets at different prediction levels.

Reference	Architecture	Dataset	HTR Level
[64]	Grapheme-based MLP-HMM + Gaussian Mixture HMM + MDLSTM-RNN	RIMES	Word and multi-word level
[68]	Decoupled Attention Network (DAN)	IAM and RIMES	Word level
[72]	Deep Convolutional Network + Recurrent Encoder-Decoder Network	IAM and RIMES	Word level
[65]	MDLSTM + RNN + CTC	IAM and RIMES	Line level
[74]	CNN + 1D-LSTM + CTC	IAM and RIMES	Line level
[66]	MDLSTM + Covolution Layers + FCN + CTC	IAM, RIMES 2011 and OpenHaRT	Line level
[91]	MDLSTM + CTC	IAM, RIMES and OpenHaRT	Line level
[67]	Attention-based RNN + LSTM	RIMES	Line level
[73]	CNN + BLSTM + S2S + CTC	IAM, RIMES and StAZH	Line level
[75]	Gated-CRNN	IAM and RIMES	Paragraph level
[80]	Transformer joint	ICDAR 2017 Esposalles and FHMR	Paragraph level
[78]	Simple Predict & Align Network (SPAN)	RIMES, IAM and READ 2016	Paragraph level
[79]	Vertical Attention Network (VAN)	RIMES, IAM and READ 2016	Paragraph level
[83,84]	Document Attention Network (DAN)	RIMES 2009 and READ 2016	Page level

### 2.4. Commercial Systems in Handwritten Text Recognition

There are some online HTR commercial systems available for transcribing both modern and historical text such as Transkribus [97], Ocelus, Konfuzio, and DOCSUMO. All of these systems use artificial intelligence technology to recognize the target texts in a variety of languages, including English, French, Spanish, and more. These systems can be extremely beneficial in the archives transcribing process. It is important to acknowledge that the majority of these systems demand a cost for their services. Nevertheless, this cost is frequently lower than manual transcription services cost and greatly lowers the time required for the task. Some of these systems have offered free trials of their services, where users have the opportunity to test it on a variety of handwritten text images with diverse writing styles. These HTR commercial systems could be accessed through the links summarized in Table 3.

We provided an overview of the state-of-the-art in HTR systems, focusing on recent surveys, advancements in recognition methodologies, and commercial solutions. Various HTR architectures were employed for the recognition of different languages at various prediction levels. These levels range from character and word recognition to line, paragraph, and document recognition.

The selection of an appropriate architecture is frequently depends on the characteristics of the target language and dataset. The following section presents some freely available modern and historical datasets that could be utilized to improve the performance of HTR systems.

## 3. Datasets

In recent decades, various handwritten text dataset images have been utilized for both modern and historical documents in different languages to enhance the recognition rates of state-of-the-art systems, particularly those identified in the literature as benchmark datasets. Table 4 provides information about the language and year of the datasets, while Table 5 shows more detailed insights into these datasets.

### 3.1. IAM

In 1999, authors of [54] introduced Institut für Informatik und Angewandte Mathematik (IAM). This is a modern English gray-scaled handwriting benchmark dataset composed of 1539 pages of scanned text written by 657 writers with a resolution of 300 dpi, which is based on the Lancaster–Oslo/Bergen (LOB) corpus. The dataset includes about 13,353 isolated and labeled text lines with their corresponding transcriptions and consists of 79 different characters, including white space. IAM is able to train HTR models at page, paragraph, line, and word levels.

### 3.2. Washington

Washington dataset [98,99] (part of IAM Historical database) is constituted by English historical manuscripts created from George Washington’s papers from the 18th century at the Library of Congress. It has data consisting of 20 pages only, 656 text lines binarized and normalized, 4894 words, and 82 unique characters, and annotated at the word level.

### 3.3. Saint Gall

Saint Gall dataset, its also part of IAM Historical database, contains handwritten historical manuscripts in Latin from the 9th century written by a single writer; the manuscript was originally from the Abbey library of Saint Gall, Switzerland. It comprises 60-page images at a resolution of 300 dpi, 1410 text lines binarized and normalized, 11,597 words, 4890-word labels, 5436-word spellings, and 49 letters. Ground truth was created using the semi-automatic proceeding proposed in [100], including text line locations, word locations, and transcription at line level.

### 3.4. Bentham

Bentham is a collection of historical manuscripts written by English philosopher Jeremy Bentham (1748–1832) and transcribed by volunteers. It consists of legal forms and drafts in English, with a few pages in French and Latin. Additionally, a public web platform has been utilized to transcribe approximately 25,000 pages [101]. The ground truth of the dataset possesses information about the layout and the transcription at line level in page format.

### 3.5. KHATT

In 2012, authors of [43,44] produced the KFUPM Handwritten Arabic TexT (KHATT), a dataset of unconstrained handwritten Arabic text, developed by a research group from KFUPM, Dhahran, Saudi Arabia, and written by 1000 distinct writers. It includes 2000 similar text paragraph images and 2000 unique text paragraph images of 6742 extracted text line images with manually verified ground truth.

### 3.6. EPARCHOS

EPARCHOS is a Greek historical handwritten codex written by Antonius Eparchos and Camillos Zanettus [60]. It includes around 120 scanned handwritten text pages, 9285 text lines, and 18,809 words for 1500–1530, representing the most important abbreviations, logograms, and conjunctions in the Greek codex. The dataset is divided into training, validation, and testing text line sets, 1435 (63%), 381 (17%), and 456 (20%), respectively, consisting of 312 characters.

### 3.7. READ

The READ dataset is a part of the European Union’s Horizon 2020 project and was first proposed at ICFHR 2016 competition on HTR [102]. The dataset is a subset of the Ratsprotokolle collection, containing early modern German handwriting. It encompasses the minutes of council meetings held between 1470 and 1805, comprising approximately 30,000 pages. It is also available with annotations in color images at page, paragraph, and line levels.

### 3.8. Esposalles

Esposalles dataset [81] consists of colored historical handwritten records at a resolution of 300 dpi representing the marriage licenses books in the archives of the cathedral of Barcelona. It comprises 291 books with information about 600,000 unions between 1451 and 1905. Each book contains an index of approximately 29 pages (1563 text lines) with information about the husbands’ surnames, the page number, and information on the transcription text file. Approximately 170 pages include 1747 marriage records and 5500 text lines written by one writer; the records contain information about the husband, wife, parents’ names, locations, occupations, and civil statuses.

### 3.9. StAZH

The Staatsarchiv des Kantons Zürich (StAZH) is a Swiss–German dataset part of the European Union’s Horizon 2020 READ project. It comprises documents of resolutions and enactments from the cabinet and the parliament of the canton of Zurich, spanning the period from 1803 to 1882.

### 3.10. MAURDOR

The MAURDOR dataset [70] comprises 10,000 heterogeneous printed, typewritten, or manuscripts documents in French, English, or Arabic, scanned or collected from different sources and annotated manually (5000 in French, 2500 in English, and 2500 in Arabic). The current dataset contains 8129 documents out of the 10,000 in TIFF format and the annotations in XML format.

### 3.11. RIMES

Reconnaissance et Indexation de données Manuscrites et de fac similÉS (RIMES) [61] consists of French mail handwritten gray-scale images at a resolution of 300 dpi. It includes 12,723 pages of 5605 letters written by 1300 volunteers representing the mail sent to companies by fax or postal mail; there are 99 different characters in the dataset. The regions of the pages are classified into several classes: sender, recipient, subject, date, location, opening, body, and attachment. Segmentation and transcription are also available at paragraph, line, and word levels.

### 3.12. e-NDP

In 2019, the e-NDP corpus was created, consisting of the registers of Notre-Dame-de-Paris and its cloister from the late medieval period. These registers represent 14,000 pages of the institution minutes held by the canons of the cloister weekly. The registers were written in Latin using a family of cursive scripts. In order to digitize these pages, historians transcribed a total of 500 pages from 26 registers dating between 1326 and 1504 to train HTR models for transcription purposes. Additionally, a specific page layout with stereotypical patterns (e.g., titles, names, and margin notes) assists in the transcription process.

### 3.13. HOME-Alcar

In 2021, Institut de Recherche et d’Histoire des Textes (IRHT-CNRS) introduced the HOME-Alcar corpus by the European research project History Of Medieval Europe (HOME). This corpus includes manuscript images with their scholarly editions at line level in addition to the annotation of named entities (e.g., persons’ names and places) to train HTR models. It consists of 17 French cartularies, such as the medieval documents of the 12th and 14th centuries, around 3090 acts, with 2760 in Latin and 330 in old French written using different script families Textualis, Cursiva Antiquior, and Semi-Hybrida.

### 3.14. HIMANIS Guérin

The training dataset (HIMANIS Guérin) was released and made available on Zenodo. It consists of 1500 images and 30,000 text lines of ground truth written in Latin and old French using a Cursiva script. These materials represent the cartulary registers produced by the French royal chancery between 1302 and 1483, and contains letters of remission, mandates, ennoblements, and amortizations.

### 3.15. FHMR

**Figure 3:**
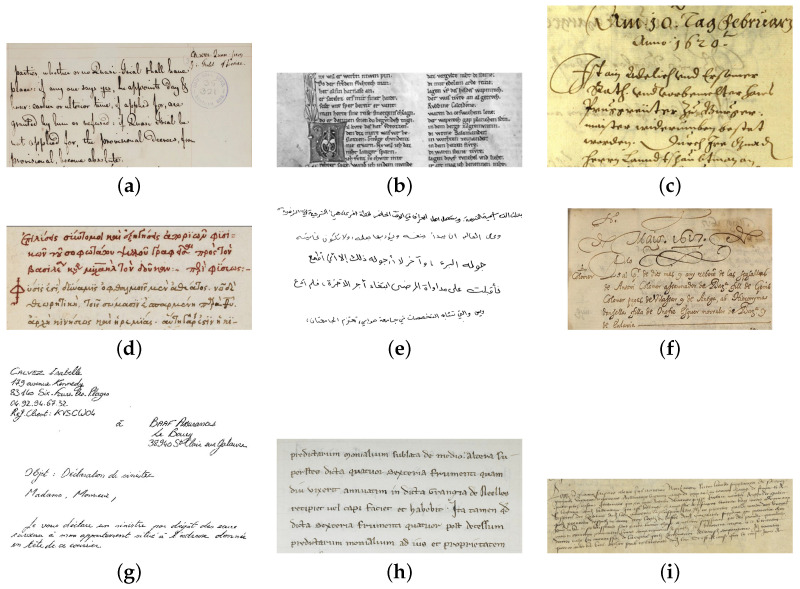
Samples of handwritten text datasets written in French and other languages: (**a**) Bentham (English). (**b**) Saint Gall (Latin). (**c**) READ (German). (**d**) EPARCHOS (Greek). (**e**) KHATT (Arabic). (**f**) Esposalles (Spanish). (**g**) RIMES (French). (**h**) HOME-Alcar (French). (**i**) HIMANIS Guérin (French).

French Handwritten Marriage Records (FHMR) [82] is a private dataset containing information concerning the husband, wife, parent’s names, occupations, and locations. It provides text images at line and paragraph levels. Figure 3 shows image examples of the datasets. Also, the presented datasets could be accessed through the links in Table 6.

### 3.16. ICDAR and ICFHR Competitions

International Conference on Document Analysis and Recognition (ICDAR) and International Conference on Frontiers in Handwriting Recognition (ICFHR) are held each year, respectively. The latter ICFHR [102,104] was conducted for the first time as an international Workshop on Frontiers of Handwriting Recognition (IWFHR) in Montreal in 1990. Then, the name changed to ICFHR in the year 2008. To date, seventeen conference editions have been held every two years. Similarly, ICDAR [103,105] has been held every two years since 1991.

Both conferences held competitions on historical document recognition using subsets of different languages dataset such as Bentham, READ, RASM, KHATT, and more. For example, ICDAR 2017 competition was for a full-page HTR. The dataset collected from the Alfred Escher Letter Collection (AEC), written in German, French, and Italian, consists of two training sets. The first includes 50 fully annotated images with their corresponding transcription ground truth. In contrast, the second set includes 10,000 images with annotated line breaks. Table 7 exhibits examples of these two competitions and the winner architecture. The competitions datasets could be accessed through the links in Table 8.

### 3.17. HTR-United

HTR-United is an online ecosystem hosted on Github that comprises a catalog of datasets and, eventually, models for handwritten text recognition. The developers of a wide range of datasets have made their work available in this catalog, together with the corresponding ground truth data. The portal allows users to download these datasets or contact their owners. The catalog aggregates metadata detailing the content of the datasets, including temporal factors, the number of contributors, and distinguishing qualities, in addition to enabling access to the data. It also contains information about the circumstances behind the datasets’ creation, such as the controlling authority, annotation criteria, and data format. The catalog is available online at the following link: https://htr-united.github.io/ (accessed on 15 November 2023)

### 3.18. The Belfort Civil Registers of Births

The proposed civil registers of births in the commune of Belfort, spanning from 1807 to 1919, comprises 39,627 birth records at a resolution of 300 dpi. These records were chosen for their homogeneity, as they feature Gregorian dates of birth starting from 1807 and are available until 1919 due to legal reasons.

**Figure 4:**
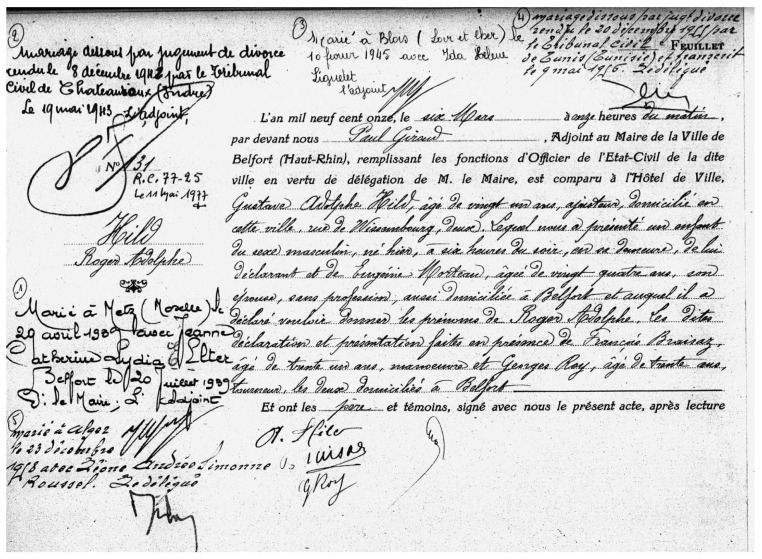
Sample of Belfort civil registers of births, featuring a hybrid mix of printed and handwritten text, along with marginal annotations.

The registers initially consist of completely handwritten entries, later transitioning to a partially printed format with spaces left free for the individual information concerning the declaration of the newborn. The transition to this hybrid preprint/manuscript format varied from one commune to another. In Belfort, it occurred in 1885 and concerns 57.5% of the 39,627 declarations. The record contain crucial information, including the child’s name, parent’ names, witnesses, among other relevant data. Figure 4 provides a visual representation of a sample page from the civil registers, while Table 9 outlines the structure and content of an entry within the archive.

The archive is publicly accessible online until the year 1902 via the following link: https://archives.belfort.fr/search/form/e5a0c07e-9607-42b0-9772-f19d7bfa180e (accessed on 12 November 2023). Additionally, we have obtained permission from the municipal archives to access data up to the year 1919.

#### 3.18.1. Belfort Records Transcription Challenges

Belfort records pose several challenges that complicate the transcription process of its entries, categorized into seven main areas:

- Document layout: The Belfort registers of birth exhibit two document layouts. The first type consists of double pages with only one entire entry on each page, while the second type comprises double pages with two entire entries per page. Each entry within these layouts contains the information outlined in Table 9. However, there are some documents where entries begin on the first page and extend to the second page.
- Reading order: It is important to identify the reading order of text regions, including the main text and marginal annotation text within the entry.
- Hybrid format: Some of the registers consists of entries that includes printed and handwritten text, as shown in Figure 4.
- Marginal mentions: These mentions pertain to the individual born but are added after the birth, often in different writing styles and by means of scriptural tools that can be quite distinct. Moreover, they are placed in variable positions compared to the main text of the declaration.
- Text styles: The registers are written in different handwritten styles that consist of angular, spiky letters, varying character sizes, and ornate flourishes, resulting in overlapped word and text lines within the script.
- Skewness: Skewness refers to the misalignment of handwritten text caused by human writing. Many handwritten text lines in the main paragraphs and margins exhibit variations in text skew, including vertical text (90 degrees of rotation). Effective processes are needed to correct the skewness of the images for any degree of rotation.
- Degradation: The images exhibit text degradation caused by fading handwriting and page smudging (ink stains and yellowing of pages).

In this section, we have presented saveral modern and historical HTR datasets from various languages and time periods, especially those identified as benchmark datasets in ICFHR and ICDAR competitions. The datasets exhibit variations in writing styles and document layouts, offering a rich set of challenges for future research directions.

Additionally, we highlighted the importance of Belfort civil registers of births in advancing the field of handwritten text recognition due to its impediments in document layouts, reading orders, hybrid formats, marginal mentions, diverse text styles, skewness, and degradation. However, overcoming these challenges requires innovative approaches in image processing, text recognition, and layout analysis.

The following section presents state-of-the-art architecture stages and performance accuracy reported on the French dataset (RIMES), along with an accuracy comparison of HTR commercial systems in recognizing English and French languages.

## 4. Results

We presents a comparison of state-of-the-art methods based on French RIMES dataset using Character Error Rate (CER) and word Error Rate (WER) metrics as reported in the publications. This dataset has emerged as a benchmark in the field of handwritten text recognition, many models have been evaluated using this dataset, making it a widely accepted and recognized standard for assessing the performance of such systems. Utilizing the RIMES dataset allows for meaningful and relevant comparisons, ensuring that our research facilitates more accurate assessments of systems performance and highlights the best approaches in this field.

Additionally, we evaluated some of the HTR commercial systems using our proposed dataset and compared their performance with that on an English dataset.

### 4.1. Evaluation Metrics

The standard and common evaluation metrics for measuring the performance of HTR systems are the Word Error Rate (WER) and the Character Error Rate (CER). WER is derived from the Levenshtein distance [108] and is defined simply as the number of errors in the recognized words divided by the total original words. Calculating the recognized words’ error includes adding substitutions, deletions, or insertions to match the recognized words and the ground truth word, as shown in the following formula:

(1) $$\begin{array}{c} WER=\frac{S+D+I}{N}=\frac{S+D+I}{S+D+C} \end{array}$$

where *S* is the number of substitutions, *D* is the number of deletions, *I* is the number of insertions, *C* is the number of correct words, and *N* is the number of words in the reference (N=S+D+C). For example, a substitution occurs when the word “French" is transcribed as “Erench”, and an insertion occurs when a new word(s) is added instead of the original, such as “work” added as “wow rock”. Finally, deletion occurs when a word is deleted from the original text, such as “bring it here” becomes “bring here”. Furthermore, CER is defined similarly but at the character level instead of the word level. A lower CER/WER result means better accuracy in the text recognition process.

### 4.2. Advances in Model Performance: State-of-the-Art Results

Experiments on multi-word recognition were carried out in [64] in three stages: word segmentation, word recognition, and language modeling. The first stage relies on the spaces between words to perform word segmentation. The second stage involves calculating the weight of each segmented word to provide N-Best recognition candidates. Finally, a scaling factor is utilized to compensate for the variation between the recognition log probabilities and model log probabilities.

A dataset consisting of 51,739 images of ICDAR2011 (a subset of the RIMES database) was employed as the training set, and 7464 images were devoted to the validation stage. The best performance on isolated word recognition processes based on recurrent neural networks recorded an error rate of nearly 10%, whereas when combining the recognizers, the system achieved an error rate of 5%. Additionally, in the multi-word recognition process, the model was trained on 1300 images, including 6039 different words, while the last 200 images of the training set were used in the validation process. The system achieved a word error rate on the evaluation set 15.2% and 11.8% on the validation set.

In [72], authors evaluated their model using the RIMES database; 11,333 text lines were devoted for training and 778 for testing. However, 10% random samples of the training set have been dedicated to the validation set. The base model recorded a word error rate of 19.1%. Additionally, integrating Layer Normalization (LN), Focal Loss, and Beam Search with the base yielded an improved recognition rate, achieving 9.6% on the same dataset. Furthermore, authors of [73] evaluated CNN-BLSTM-S2S-CTC model and achieved CER and WER of 3.13% and 8.94%, respectively.

According to [74], convolutional and one-dimensional recurrent layers perform better or at least equivalent to MDLSTM models. Experiments were conducted on the RIMES database at the line level, with 10,203 text lines used for training, 1130 for validation, and 778 for testing. The outcomes recorded significant recognition rates, and further improvements were observed when synthetic data augmentation was used during the training stage, resulting in a CER of 2.3% and a WER of 9.6% with a word 4-gram language model, and a CER of 2.5% and a WER of 9.0% without a language model.

Authors of [75] conducted experiments demonstrating that convolutions are faster than LSTMs, and 1D LSTMs are faster than MDLSTMs. They proposed the Gated-CRNN with BLSTM model, which utilizes GPU training to enhance model speed, size, and accuracy. The experiments were conducted using the IAM and RIMES datasets at the line and paragraph levels. The results achieved a CER of 1.9%, and a WER of 7.9% at line level, and a CER of 2.2%, and a WER of 7.9% at paragraph level, without line segmentation process.

Additionally, authors of [80] conducted experiments at both the line and paragraph levels. For their research, 997 FHMR marriage records were allocated to the training set, 103 records for the validation set, and 132 records for the evaluation set. To determine the best hyper-parameters for the proposed model, they employed a joint tagging encoding technique of the ground truth and conducted an ablation study. The ablation study revealed that the experimental outcomes improved by excluding experiments on images larger than 384 × 1024 pixels. Authors reported that the best performance was achieved using images of size 256 × 1024 pixels at both the paragraph and line levels. They also concluded that 2D positional encoding performs better than 1D positional encoding at both levels. Furthermore, the integration of the “<eol>” label in the ground-truth transcription resulted in improved accuracy rate. The model’s outcomes demonstrated high recognition quality when utilizing Transformer and joint two-stage mixed-level training. In fact, they outperformed other architectures applied to FHMR images at the paragraph level (marriage record).

The Simple Predict & Align Network (SPAN) proposed in [78] was tested on the RIMES dataset at both the text line and paragraph levels, in which 1500 paragraphs were devoted to the training set, and 100 paragraphs assigned to the evaluation set. The last 100 training images were utilized as the validation set. In order to mitigate model overfitting, data augmentation techniques were employed during the model training process. These techniques included image resolution modification, perspective transformation, elastic distortion, dilation and erosion, and brightness adjustments. The experimental results were compared with other methods and demonstrated competitive performance in terms of CER and WER metrics. Furthermore, it was observed that pre-training the model at the line level significantly improved the recognition task. They also designated Vertical Attention Network (VAN) for handwritten paragraph text recognition [79]. Experiments conducted on the RIMES dataset using the same training, evaluation, and validation sets as mentioned previously. It involved segmentation and transcription at both the paragraph and line levels. Moreover, data augmentation techniques and post-processing steps were applied. This involved employing CTC best path decoding to identify the last character within the white space character probabilities lattice and subsequently removing it if it was found to be redundant or localized either at the beginning or the end of the text line. VAN was pre-trained on text line images using the learned-stop strategy. The results achieved a CER of 1.91%, and a WER of 6.72%.

Recently, the Document Attention Network (DAN) [83] was evaluated for text recognition and document layout analysis using RIMES 2009 dataset at page and paragraph levels, where 1050 pages were allocated for the training set, 100 pages for the validation set, and 100 pages for the testing set. Additionally, the model was tested using the RIMES 2011 dataset (1400 for training, 100 for validation, and 100 for testing) at paragraph and line levels to compare the performance with other pre-segmented input architectures.

Furthermore, to address the lack of training data, synthetic documents were generated to facilitate the learning of document reading. An ablation study was conducted on the test set of the dataset at single-page and double-page levels with a two-day training period. The model was subjected to various experiments wherein each training component was independently removed. The results obtained from these experiments demonstrated significantly higher accuracy rates compared to other handwritten text recognition approaches. However, the CER percentage may be inaccurate due to misreading order when compared with the ground truth.

**Figure 5:**
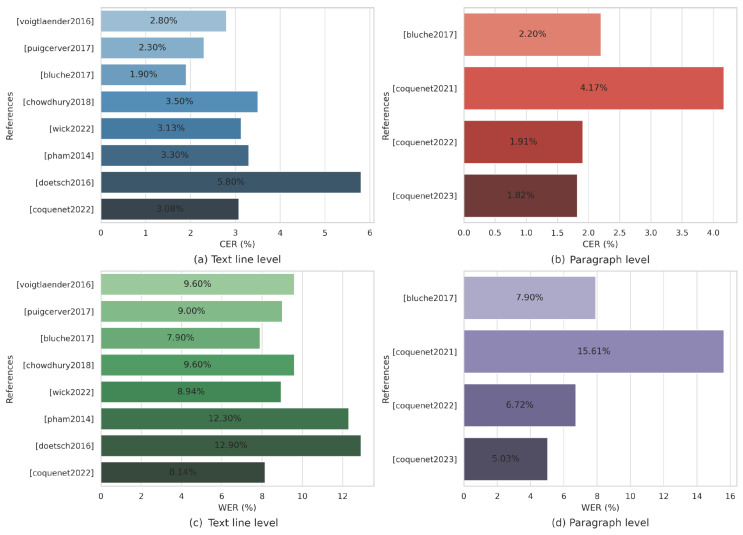
State-of-the-art Character Error Rate (CER) and Word Error Rate (WER) across various studies applied to the French language at two different levels: text line and paragraph. (**a**) Shows the CER at the text line level, based on studies by Voigtlaender et al. [65], Puigcerver et al. [74], Bluche et al. [75], Chowdhury et al. [72], Wick et al. [73], Pham et al. [91], Doetsch et al. [67], and Coquenet et al. [79]. (**b**) Depicts the CER at the paragraph level, as reported in studies by Bluche et al. [75], Coquenet et al. [78,79,83]. (**c**,**d**) Present the WER at the text line and paragraph levels, respectively, from the same studies.

Additionally, two metrics have been proposed to evaluate the layout recognition process in handwritten document recognition. First, the Layout Ordering Error Rate (LOER) assesses the accuracy of layout recognition. Second, the mean Average Precision Character Error Rate (mAPCER) is employed to evaluate the recognition of the document layout with respect to the text content. Although the model has shown improvements over the state-of-the-art outcomes, the prediction time is still high, recording linear growth with the number of tokens to be predicted. Figure 5 depicts the CER and WER of state-of-the-art methods at the line and paragraph levels. Table 10 presents a comparison of state-of-the-art methods’ CER and WER accuracies based on the French RIMES dataset.

The research was extended in [84] to improve the prediction time of the model through two-step strategy. First, the model predicts the first character of each text line within the page. Second, it predicts all the text lines in parallel using multi-target queries and a document positional encoding scheme. Three document-level datasets have been used to evaluate the speed of the new model, and the findings indicate that the new DAN [84] achieved a faster prediction time, reaching a speed four times faster (1.4 s compared to 5.6 s for the initial DAN). Additionally, the approach demonstrated competitive recognition rates compared to DAN [83] and other state-of-the-art approaches. It is worth mentioning that the models [83,84] were also tested using other language datasets such as READ 2016 and MAURDOR dataset.

Table 11 illustrates the development years of the models. Furthermore, Table 12 displays the training, validation, and testing sets of the French datasets utilized by the state-of-the-art techniques.

#### State-of-the-Art Techniques Limitations

It is worth noting that limited resources are available for the French historical datasets with their transcriptions. Hence, minimal research has been conducted on such documents. The lack of common benchmarks for historical French texts adds impediments to objectively comparing the performance of various handwritten text recognition systems.

Consequently, most of the studies have been conducted on modern French dataset, such as RIMES, which are specified as standard benchmark dataset in ICFHR and ICDAR competitions. These datasets are commonly used to validate state-of-the-art approaches in the recognition of handwritten French text.

### 4.3. Commercial HTR Systems

**Figure 6:**
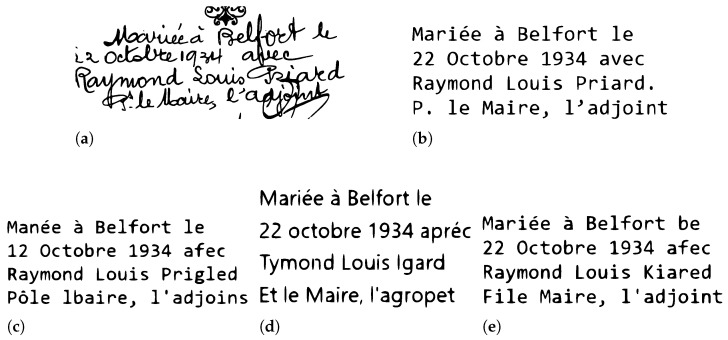
Examples of using Ocelus, Transkribus, and DOCSUMO on a margin segment from Belfort civil registers of births (historical French handwritten text): (**a**) The original text image. (**b**) The original text ground truth. (**c**) The result of utilizing Ocelus. (**d**) The result of utilizing Transkribus. (**e**) The result of utilizing DOCSUMO.

**Figure 7:**
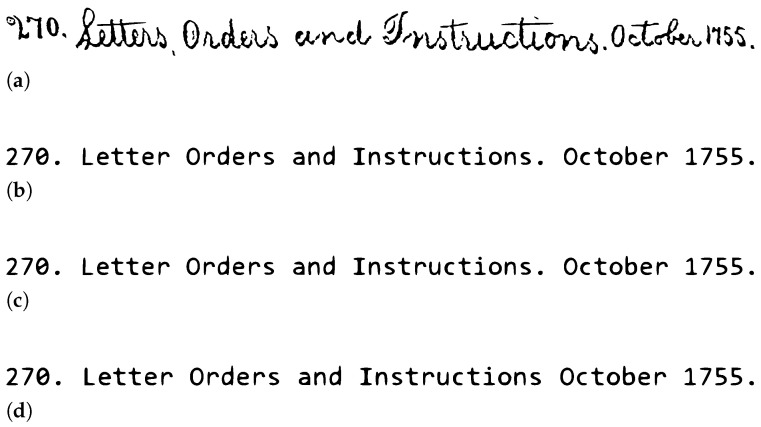
Examples of using Ocelus, Transkribus, and DOCSUMO on Washington dataset (historical English handwritten text): (**a**) The original text image. (**b**) The original text ground truth. (**c**) The result of utilizing Ocelus and DOCSUMO. (**d**) The result of utilizing Transkribus.

To evaluate the effectiveness of commercial systems in recognizing handwritten text in both English and French languages, three systems: Ocelus, Transkribus, and DOCSUMO were chosen to conduct this experiment, as they are among the most well-known and offer free trials for text recognition. For the evaluation, a text line image of Washington dataset was utilized for the English language, and a margin segment from the proposed Belfort civil registers of births was used for the French language. These experiments allowed us to compare and demonstrate the performance of these systems on the recognition of different international languages and various handwriting styles. Figure 6 and Figure 7 display examples of the results obtained from Ocelus, Transkribus, and DOCSUMO. Table 13 provides a detailed comparison of their performance.

In this section, we have presented an overview of the evaluation stage of HTR models and commercial systems, utilizing metrics such as Character Error Rate (CER) and Word Error Rate (WER). We have also highlighted advances in model design and performance, providing detailed insights into experiments and outcomes from various studies conducted on the RIMES dataset. Additionally, the evaluation of commercial systems in both English and French has revealed varying degrees of accuracy, with the most beneficial performance observed in the context of the English language.

The subsequent section provides a discussion of the optimal architecture for transcribing historical documents, encompassing diverse challenges, including those posed by the French Belfort civil registers of births.

## 5. Discussion

This study surveys several interesting state-of-the-art techniques, and that mainly focus on recognizing French handwritten text. Table 2 illustrates the techniques utilized at each recognition level (word, text line, paragraph, page), where most of the studies concentrated on word and text line levels. Notably, the majority of researchers relayed on LSTMs with CTC systems to fulfill the objectives since the LSTMs networks appear to provide the highest performances in solving high-dimensional dataset situations as compared to other techniques, particularly during the period from 2012 to 2020. This trend is depicted in Table 11, which summarizes the year-wise distribution of these techniques. However, it is worth noting that this addressed architecture still holds expensive computational costs.

Moreover, it is shown that minimal recent studies conducted between 2021 and 2023 have focused on paragraph and page levels using the attention mechanism. As illustrated in Figure 5, the best-performing architecture at the text line level achieved CER of 1.9%, authors of [74] verified that convolutional and one-dimensional recurrent layers perform better than MDLSTMs models in terms of CER metrics. Additionally, the attention mechanism proposed in [83] yielded the highest performance for the WER metric, achieving an impressive 6.78%. The latter also outperformed the state-of-the-art at the paragraph and page levels, as shown in Table 10.

According to the historical datasets detailed in Table 5, it is noticed that limited resources are available for the French historical datasets with their transcriptions. Thus, researchers attempt to devote a data augmentation process to overcoming this limitation. Additionally, they encountered critical challenges within the historical dataset images, such as angular, spiky letters, and ornate flourishes. These challenges result in overlapped word and text lines within the script of different handwritten text styles, leading to highest time consumption for the pre-processing phase, and strengthen the challenges over researchers attempts. Therefore, the demand arises for new considerable transcripted datasets that capture the specificities of such historical sources.

Furthermore, recognizing and digitizing such French historical datasets should be preceded by transcribing and cross-checking processes in order to enhance model development and evaluation processes. Two options can be employed for transcribing historical documents.

Firstly, manual transcription can be performed by teams or by a call to the community. However, it is time-consuming and costly. Additionally, the transcriptions produced by different transcribers may not be uniform, particularly concerning abbreviations, which may result in poor model performance.

Secondly, automatic transcription using commercial systems, as summarized in Table 3. The performance outcomes of these systems, displayed in Figure 6 and Table 13, indicate better performance on historical English language samples in comparison with French language samples. Nevertheless, these systems facilitates an increase in transcription accuracy by permitting instant manual corrections on the transcribed text, which assist in terms of vocabulary (the lexicon). It is worth mentioning that evaluating such commercial systems at a documents level has resulted in improved accuracy rates due to differences in character and word counts.

Additionally, it is also important for researcher to possess language skills when working with historical French documents. This includes a solid understanding of French grammar, vocabulary, spelling, and an awareness of writing style variations.

In response to our research question, the automatic transcription of the Belfort civil registers of births shown in Figure 4 and described in Table 9, as well as other historical documents with similar characteristics, appears to be applicable using a novel attention mechanism technique as employed in [83,84], along with additional pre-processing stages for segmenting the dataset into paragraphs and text line levels. nonetheless, manual transcription of some registers should be performed to train the model before initiating the automatic transcription process. Additionally, we might also consider employing commercial systems for this purpose, with manual corrections and associated costs. However, this archive constitutes many challenges, as in Section 3.18.1, offering rich opportunities for enhancing researchers’ models and historical research.

## 6. Suggestions for Future Directions

The field of historical handwritten text recognition poses challenging research problems due to various complications that still need to be addressed. The following are some suggestions for future research directions that could enhance the accuracy of the model recognition:

- Pre-processing: Developing an effective approach based on machine learning techniques for historical text skew detection and correction, text degradation, and document layout analysis could highly improve the recognition accuracy of the models.
- Segmentation: Implement an additional process after the segmentation stage to refine the segments and preserve the shape of historical handwritten text.
- Classification models: Combining various classifiers to handle the recognition of hybrid-form documents, in other words, documents that include printed and historical handwritten text. This also involves utilizing different optimizers within the model to improve the accuracy rate.
- Post-processing: Extend the existing post-processing techniques to capture semantic relationships between words and reduce errors by excluding less reliable predictions.

## 7. Conclusions

Handwritten text recognition systems have made significant progress in recent years, becoming increasingly accurate and reliable. In this study, we have presented several state-of-the-art models and achievements in offline handwritten text recognition across various international language documents. Additionally, we presented a comprehensive survey of French handwritten text recognition models specifically. The research papers were reviewed at four HTR levels: word, text line, paragraph, and page. Furthermore, we provided a summary of available public datasets for both French and other languages.

Despite significant achievements in recognizing modern handwritten text, there is still a need to extend these capabilities to historical text documents. Historical handwritten text recognition poses unique challenges, such as transcription cost, a variety of writing styles, abbreviations, symbols, and reproduction quality of historical documents.

We also observed that some commercial handwritten text recognition systems are performing exceptionally on handwritten text in English. In contrast, they are inaccurate in recognizing the French historical cursive handwritten text. Nevertheless, these systems could be a promise tool that can assist in automatically transcribing large volumes of historical documents with manual corrections. This is attributed to their advantages in automatic segmentation and dictionary support. Hence, decreasing time and cost.

Finally, we facilitate researchers in identifying the appropriate technique or datasets for further research on both modern and historical handwritten text documents. Furthermore, we conclude that there is a compelling need to design a new technique specifically tailored for transcribing the French Belfort civil registers of births.

## Figures and Tables

**Table 1:** State-of-the-art architectures utilized for handwritten text recognition of different international Languages datasets such as English, Arabic, Russian, and others at different prediction levels.

Reference	Architecture	Dataset	HTR Level
[38]	AHCR-DLS (2-CNN)	HMBD, CMATER and AIA9k	Character level
[42]	Transformer-T and Transformer with Cross-Attention	KHATT	Character (Subword) level
[46]	Light Transformer	IAM	Character level
[53]	Attention-Gated-CNN-BGRU	Kazakh	Character level
[59]	CRNN-MDLSTM	IAM and George Washington	Line level
[60]	OctCNN-BGRU	EPARCHOS, IAM and RIMES	Line level
[15]	CRNN-FCNN	EPARCHOS, IAM and RIMES	Line level
[62]	OrigamiNet	IAM ICDAR 2017	Page level

**Table 3:** List of HTR commercial systems and their corresponding links.

Name	Link
Transkribus	https://readcoop.eu/transkribus/ (accessed on 21 November 2023)
Ocelus	https://ocelus.teklia.com/ (accessed on 21 November 2023)
Konfuzio	https://konfuzio.com/en/document-ocr/ (accessed on 21 November 2023)
DOCSUMO	https://www.docsumo.com/free-tools/online-ocr-scanner (accessed on 21 November 2023)

**Table 4:** Languages and year information of the datasets.

1999	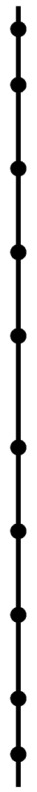	IAM (English) [54]
2001		Washington (English) [98,99]
2006		Saint Gall (Latin) [100] RIMES (French) [61]
2012		KHATT (Arabic) [43,44]
2013		Esposalles (Spanish) [81] MAURDOR (French, English and Arabic) [70]
2016		Bentham (English) [101] READ (German) [102,103]
2017		HIMANIS Guérin(French) [77]
2019		e-NDP (French) StAZH (Swiss-German)
2020		EPARCHOS (Greek) [60]
2021		HOME-Alcar (French)

**Table 5:** Datasets details: total number of pages, lines, and words.

Dataset	Language	Total Pages	Total Lines	Total Words
IAM	English	1539	13,353	115,320
Bentham	English	25,000	-	-
Washington	English	20	656	4894
KHATT	Arabic	2000 Paragraph	6742	-
EPARCHOS	Greek	120	9285	18,809
READ	German	30,000	-	-
Saint Gall	Latin	60	1410	11,597
Esposalles	Spanish	199	7063	-
MAURDOR (HT)	French, English, and Arabic	8129	49,412	-
RIMES	French	1500	12,723	-
HOME-Alcar	French	330 acts	-	-
e-NDP	French	500	33,735	-
HIMANIS Guérin	French	1500	30,000	-

**Table 6:** List of the datasets and their corresponding links.

Dataset	Link
IAM	https://fki.tic.heia-fr.ch/databases/iam-handwriting-database (accessed on 14 October 2023)
Bentham	https://zenodo.org/record/44519#.Y_h6fx9ByMo (accessed on 14 October 2023)
Washington	https://paperswithcode.com/dataset/george-washington (accessed on 14 October 2023)
KHATT	http://khatt.ideas2serve.net/KHATTDownload.php (accessed on 14 October 2023)
EPARCHOS	https://zenodo.org/record/4095301#.Y_eylx9ByMo (accessed on 17 October 2023)
READ	https://zenodo.org/record/218236#.WnLhaCHhBGF (accessed on 17 October 2023)
Saint Gall	https://fki.tic.heia-fr.ch/databases/saint-gall-database (accessed on 17 October 2023)
Esposalles	http://dag.cvc.uab.es/the-esposalles-database/ (accessed on 17 October 2023)
StAZH	https://archives-quickaccess.ch/stazh (accessed on 18 October 2023)
MAURDOR	https://catalog.elra.info/en-us/repository/browse/ELRA-E0045/ (accessed on 18 October 2023)
RIMES	https://paperswithcode.com/dataset/rimes (accessed on 14 October 2023)
e-NDP	https://digital.library.unt.edu/ark:/67531/metadc1477117/ (accessed on 19 October 2023)
HOME-Alcar	https://zenodo.org/record/5600884#.Y_eqoB9ByMo (accessed on 19 October 2023)
HIMANIS Guérin	https://zenodo.org/record/5535306#.Y_emnx9ByMo (accessed on 19 October 2023)
FHMR	Private

**Table 7:** Examples of ICDAR and ICFHR competitions: years, datasets, and winning techniques.

Competition	Dataset	Winner	Winner Approach
ICFHR 2014	Bentham	[106]	CRNN+lexicon
ICDAR 2015	TranScriptorium	[105,106]	CRNN B1&B2
ICFHR 2016	READ	[102]	CRNN+char10-gram
ICDAR 2017	READ	[103]	CRNN+char10-gram
ICFHR 2018	RASM, READ and others	[107]	STPP-PHOCNet

**Table 8:** Links to ICDAR and ICFHR competitions datasets.

Competition	Link
ICFHR 2014	http://doi.org/10.5281/zenodo.44519 (accessed on 24 October 2023)
ICDAR 2015	http://doi.org/10.5281/zenodo.248733 (accessed on 24 October 2023)
ICFHR 2016	http://doi.org/10.5281/zenodo.1164045 (accessed on 24 October 2023)
ICDAR 2017	http://doi.org/10.5281/zenodo.835489 (accessed on 24 October 2023)
ICFHR 2018	http://doi.org/10.5281/zenodo.1442182 (accessed on 24 October 2023)

**Table 9:** The structure of an entry in the Belfort civil registers of births.

Structure	Content
Head margin	Registration number.
	First and last name of the person born.
Main text	Time and date of declaration.
	Surname, first name and position of the official registering.
	Surname, first name, age, profession and address of declarant.
	Sex of the newborn.
	Time and date of birth.
	First and last name of the father (if different of the declarant).
	Surname, first name, status (married or other), profession (sometimes) and address (sometimes) of the mother.
	Surnames of the newborn.
	surnames, first names, ages, professions and addresses (city) of the 2 witnesses.
	Mention of absence of signature or illiteracy of the declarant (very rarely).
Margins (annotations)	Mention of official recognition of paternity/maternity (by father or/and mother): surname, name of the declarant, date of recognition (by marriage or declaration).
	Mention of marriage: date of marriage, wedding location, surname and name of spouse.
	Mention of divorce: date of divorce, divorce location.
	Mention of death: date and place of death, date of the declaration of death.

**Table 10:** Accuracy comparison (%) of state-of-the-art HTR methods on a French-language dataset, based on dataset used and recognition level. Improvement: adding classifier, feature extraction, and segmentation columns to discuss relevant previous research in greater detail.

Dataset	Ref.	Classifier	Feature Extraction	Segme- ntation	CER (%)	WER (%)	Level
RIMES 2006	[65]	MDLSTM	Automatic	No	2.8	9.6	Line
RIMES 2006	[74]	CNN + 1D-LSTM	Automatic	No	2.3	9.0	Line
RIMES 2006	[75]	GCRNN	Convolutional gates encoder	Yes	1.9	7.9	Line
RIMES 2006	[72]	Deep CN + Recurrent Encoder-Decoder Network	CNN + Sequence Learning	Yes	3.5	9.6	Word
RIMES 2006	[73]	CNN + BLSTM + S2S + CTC	CNN	No	3.13	8.94	Line
RIMES 2006	[75]	GCRNN	Convolutional gates encoder	No	2.2	7.9	Paragraph
RIMES 2009	[68]	Decoupled text decoder	CNN encoder	No	2.7	8.9	Word
RIMES 2009	[64]	Grapheme-based MLP-HMM + Gaussian Mixture HMM + RNN	Multiple methods	Yes	-	4.82	Multi Word
RIMES 2009	[83]	Transformer decoder	FCN encoder	No	5.46	13.04	Paragraph
RIMES 2009	[83]	Transformer decoder	FCN encoder	No	4.54	11.85	Page
RIMES 2009	[84]	Multi-target transformer	FCN encoder	No	6.38	13.69	Page
RIMES 2011	[67]	Attention-based LSTM	Automatic	No	2.9	6.8	Word
RIMES 2011	[64]	Grapheme-based MLP-HMM + Gaussian Mixture HMM + RNN	Multiple methods	Yes	-	4.75	Multi Word
RIMES 2011	[91]	MDLSTM + CTC	Automatic	No	3.3	12.3	Line
RIMES 2011	[67]	Attention-based LSTM	Automatic	No	5.8	12.9	Line
RIMES 2011	[79]	Hybrid attention mechanism	FCN encoder	No	3.08	8.14	Line
RIMES 2011	[83]	Transformer decoder	FCN encoder	No	2.63	6.78	Line
RIMES 2011	[78]	Single convolutional layer + CTC	FCN encoder	No	4.17	15.61	Paragraph
RIMES 2011	[79]	Hybrid attention mechanism	FCN encoder	No	1.91	6.72	Paragraph
RIMES 2011	[83]	Transformer decoder	FCN encoder	No	1.82	5.03	Paragraph

**Table 11:** State-of-the-art HTR techniques applied to the French language from 2012 to 2023.

Year	2012	2014	2016	2017	2018	2020	2021	2022	2023
	[64]	[66]	[65]	[74]	[72]	[68]	[78]	[73]	[83]
**Reference**		[91]	[67]	[75]		[16]		[80]	[84]
								[79]	

**Table 12:** Details of training, validation, and testing sets in French datasets used by state-of-the-art HTR techniques.

Reference	Dataset	Training Set	Validation Set	Testing Set	Level
[65]	RIMES 2006	11,279	-	778	Line
[74]	RIMES 2006	10,203	1130	778	Line
[72]	RIMES 2006	10,203	1130	778	Line
[73]	RIMES 2006	10,171	1162	778	Line
[83]	RIMES 2009	5875	540	559	Paragraph
[84]	RIMES 2009	1050	100	100	Paragraph
[83]	RIMES 2009	1050	100	100	Page
[67]	RIMES 2011	11,275	1,128	778	Line
[83]	RIMES 2011	10,530	801	778	Line
[91]	RIMES 2011	1400	100	100	Line
[64]	RIMES 2011	1300	200	100	Paragraph
[78]	RIMES 2011	1500	100	100	Paragraph
[79]	RIMES 2011	1500	100	100	Paragraph
[83]	RIMES 2011	1400	100	100	Paragraph
[80]	FHMR	997	103	132	Paragraph

**Table 13:** Accuracy comparison (%) of HTR commercial systems on French- and English-language datasets.

	RIMES		Washington	
System	CER (%)	WER (%)	CER (%)	WER (%)
Ocelus	15	53	2	14
Transkribus	18	33	4	29
DOCSUMO	11	33	2	14

## Data Availability

The datasets (Belfort civil registers of births) analyzed during the current study are available online as mentioned, and from the corresponding author on reasonable request.

## Abbreviations

The following abbreviations are used in this manuscript:

DL	Deep Learning
HTR	Handwritten Text Recognition
OCR	Optical Character Recognition
HMM	Hidden Markov Model
CNN	Convolutional Neural Networks
FCN	Fully Convolutional Network
RNN	Recurrent Neural Networks
CRNN	Convolutional Recurrent Neural Networks
BGRU	Bidirectional Gated Recurrent Units
LSTM	Long Short Term Memory
MDLSTM	Multi Dimensional Long Short Term Memory
BLSTM	Bidirectional Long Short Term Memory
S2S	Sequence-To-Sequence
CTC	Connectionist Temporal Classification
GPU	Graphics Processing Unit
LOER	Layout Ordering Error Rate
mAPCER	mean Average Precision Character Error Rate
mAP	mean Average Precision
CER	Character Error Rate
WER	Word Error Rate
XML	Extensible Markup Language
MLP	Multi-Layer Perceptron
